# On the Dynamics of International Real-Estate-Investment Trust-Propagation Mechanisms: Evidence from Time-Varying Return and Volatility Connectedness Measures

**DOI:** 10.3390/e23081048

**Published:** 2021-08-14

**Authors:** Keagile Lesame, Elie Bouri, David Gabauer, Rangan Gupta

**Affiliations:** 1 Department of Economics, University of Pretoria, Pretoria 0002, South Africa; keagilel@gmail.com; 2 Adnan Kassar School of Business, Lebanese American University, Beirut 13-5053, Lebanon; eliebouri@usek.edu.lb; 3 Data Analysis Systems, Software Competence Center Hagenberg, 4232 Hagenberg, Austria; david.gabauer@hotmail.com

**Keywords:** REITs, TVP-VAR, dynamic connectedness, C32, C50, G10

## Abstract

In this paper, we investigate the time-varying interconnectedness of international Real Estate Investment Trusts (REITs) markets using daily REIT prices in twelve major REIT countries since the Global Financial Crisis. We construct dynamic total, net total and net pairwise return and volatility connectedness measures to better understand systemic risk and the transmission of shocks across REIT markets. Our findings show that that REIT market interdependence is dynamic and increases significantly during times of heightened uncertainty, including the COVID-19 pandemic. We also find that the US REIT market along with major European REITs are generally sources of shocks to Asian-Pacific REIT markets. Furthermore, US REITs appear to dominate European REITs. These findings highlight that portfolio diversification opportunities decline during times of market uncertainty.

## 1. Introduction

International capital markets have become increasingly integrated since the 1990s as global interconnectedness and financial liberalization have expanded. As a result, a return shock in one asset market can be transmitted to another asset market. Volatility, which represents uncertainty and risk in financial markets, is also investigated to understand the transmission dynamics of volatility shocks, which helps investors manage portfolio risks and market regulators to respond effectively to its consequences. Moreover, advances in trading technology, including the rapid flow of information across international financial markets, has further led to higher return and volatility in financial markets (Zhang [1], Boehmer et al. [2]). There is a large body of empirical literature studying the transmission of return and volatility shocks across equity markets; however, evidence on the cross market transfer of these shocks in REIT markets specifically are few.

REITs have become an attractive investment vehicle for investors because they offer the benefits of investing in direct real estate such as rental profits while overcoming the high entry costs and the relatively low liquidity associated with direct real estate investment. REIT investors have access to a diversified portfolio of underlying real estate, which reduces the risk of property investment. The growth and popularity of REITs as an investment vehicle in turn provides liquidity to the real estate market, contributing to its development. The global REIT market has grown significantly to USD 1.4 trillion at the end of 2019 in nearly 40 countries since the 1960s when REITs were first established in the US (NAREIT, 2020) (https://www.reit.com/, accessed on 5 October 2020). In addition, as the global REIT trading volumes increased since their inception, REIT volatility shocks have become persistent; see Cotter and Stevenson [3], Zhou and Kang [4]. Therefore, an in-depth understanding of the spillover dynamics in international REIT markets has become increasingly important for REIT investors in particular knowledge of the sources and recipients of REIT shocks, REIT market interdependencies, as well as how they have evolved over time and during crisis periods. The objective of this paper is to study the daily dynamic return and volatility spillover or connectedness in international REITs based on a representative sample of major REIT markets (Belgium, United Kingdom, France, Germany, Japan, Netherlands, New Zealand, Canada, Australia, Hong Kong, Singapore, and the United States), with the United States being the only mature REIT market and the rest of the countries in the sample being *established* markets (EY Global 2018, How REIT regimes are doing in 2018, EY Global, viewed 5 October 2020, https://www.ey.com/en_gl/real-estate-hospitality-construction/how-reit-regimes-are-doing-in-2018, accessed on 10 September 2020).

REITs offer diversification benefits to investors because of their imperfect covariance with the broader stock market (Chandrashekaran [5], Huang and Zhong [6], Hoesli and Oikarinen [7], Anderson et al. [8], Boudry et al. [9]). This has made REITs an important asset class for portfolio allocation and diversification purposes. There is an abundance of empirical literature studying return and volatility spillovers between REITs and non-REIT equity markets; for example, see Chiang et al. [10], Damianov and Elsayed [11], and Lin [12]. The few available studies that focus on spillovers across global REITs primarily estimate variance and mean effects based on Generalized Autoregressive Conditional Heteroskedasticity (GARCH) models (Lin [12], Cotter and Stevenson [13], Pham [14], Li et al. [15], Hoesli and Reka [16]), while Zhou and Kang [4] estimated Granger causality tests to determinant the direction of volatility spillovers. (Both Pham [14] and Li et al. [15] use the exponential GARCH model (EGARCH) proposed by Nelson [17], which can be used to determine the asymmetric impact of positive and negative news on volatility. Earlier work by Cotter and Stevenson [18] studied the volatility linkages within REIT sub-sectors and between REIT markets and US equity markets using the BEKK-GARCH model developed by Engle and Kroner [19] and similarly Hoesli and Reka [16] used the BEKK model.) Anderson et al. [8] employed a range based time-varying logarithmic model CARR model (TVLCARR) to investigate volatility dynamics in the REIT markets, which has the advantage of being able to capture structural changes in volatility dynamics.

Other papers adopt the Diebold and Yılmaz [20,21] connectedness framework, which is used to estimate the time-varying interdependency and systemic risk in financial markets and the economy. Liow and Newell [22] and Liow and Huang [23] are the only two studies that used this approach to investigate international REIT market connectedness to the best of our knowledge. Specifically, Liow and Newell [22] calculated a volatility spillover index (Diebold and Yılmaz [20]), while Liow and Huang [23] employed the extended Diebold and Yılmaz [21] measure. This paper follows this framework with methodological improvements. The Diebold and Yılmaz [20] framework computes dynamic total connectedness derived from the decomposition of the forecast error variance of the familiar vector autoregressive [24]. Specifically, the forecast error variance of a variable is split into parts attributable to other variables in the system, and these cross variance shares or spillover effects are aggregated into a single index. The framework was further modified by Diebold and Yılmaz [21] to produce directional and net connectedness estimates that are invariant to the ordering of variables, as result of the Cholesky factorization, by using the generalized vector autoregressive framework of Koop et al. [25] and Pesaran and Shin [26]. The authors further showed that variance decomposition of VARs is closely related to network connectedness (Diebold and Yılmaz [27]). We estimate the return and volatility connectedness in global REIT markets based on a time-varying parameter vector autoregressive (TVP-VAR) connectedness framework of Antonakakis et al. [28]. The latter allows the variance–covariance structure to be time-varying instead of the fixed rolling window VAR estimates from the Diebold and Yilmaz framework, which were adopted by Liow and Newell [22] and Liow and Huang [23]. The TVP–VAR-based connectedness framework of Antonakakis et al. [28] integrates the TVP-VAR framework of Koop and Korobilis [29] with the connectedness framework of Diebold and Yılmaz [21,27]. The spillover measures based on the TVP-VAR connectedness approach are an improvement of the Diebold and Yılmaz [21,27] framework because there is no rolling window involved, and as a result there is no need to arbitrarily select the size of the rolling window, which could lead to parameter estimates that are not precise and a loss of observations associated with the rolling window analysis. Notably, Antonakakis et al. [28] showed that the TVP–VAR-based connectedness measures can more accurately estimate possible changes in parameter estimates, and that these measures are not sensitive to extreme outliers, which is the case with the rolling window VAR approach. Finally, our sample period covers the COVID-19 outbreak, during which REITs have been adversely affected by the pandemic. For example, global REITs contracted by 11.8% in 2020 while global stock markets closed 14.1% higher in the same year. (The S&P 500 Global REIT and the MSCI World Indices were used to measure the performance of global REITs and stock markets, respectively. The calculations are the authors’ and the data is sourced from the Bloomberg database.) The divergent performance highlights how the COVID-19 pandemic severely affected REITs despite a recovery in stock markets. Therefore, this paper also examines the dynamics of systemic risk and the transmission of shocks across REIT markets during this unprecedented pandemic.

The rest of the paper proceeds as follows. Section 2 presents the literature review followed by a discussion of the dataset Section 4. Section 3 describes in detail the employed methodology. The empirical results and their implications are discussed in Section 5, while Section 6 concludes the paper.

## 2. Literature Review

The empirical literature on the connectedness of return and volatility shocks in international REIT has primarily focused on the interdependence between REIT markets and other asset markets including general equities. For example, Cotter and Stevenson [13] studied the return and volatility linkages within US REIT sub-sectors and the influence of US equity markets on global REIT volatility. Tsai et al. [30] looked at the return or mean relationship between global REIT and equity markets. Hoesli and Reka [31] also examined the volatility spillover between REIT and stock markets, while Lin [12] extended this relationship to include spillover effects between bond markets and REITs. Liow [32]’s REIT cross-market volatility connectedness involved money, exchange rates, stocks, and bond markets. Damianov and Elsayed [11] also followed the Diebold and Yılmaz [21,27] framework but investigated spillovers between REITs, housing, mortgage, and stock markets.

The is a dearth of empirical literature focused on understanding the transmission dynamics of shocks across international REIT markets alone, and this paper seeks to contribute to this line of inquiry. Some of the available evidence is briefly summarized in the rest of this section. Earlier studies such as Zhou [33] investigated extreme volatility spillovers in six major REIT markets (United States, United Kingdom, Singapore, Australia, Hong Kong, and Japan) between1990 and 2010. The author use Value at Risk (VaR) as the volatility measure, which estimates the maximum loss a portfolio can incur and applies the Granger causality in risk procedure [34] to examine these cross market effects. The paper finds that volatility spillovers tends to run from a larger market to small market, while the bi-directional spillover risks are found only within the Asia pacific region. Additionally, both downside and upside spillover risks have become more frequent and stronger over time.

Many of the earlier studies used various extensions of the GARCH model [35], and the parameter estimates from these models were used as measures of the effects of conditional return and volatility shocks. Pham [14] examined the return and volatility cross market transmission in seven Asian REIT markets split between developed and emerging Asian REIT markets based on Exponential GARCH approach [17] over the period between 2006 and 2011. The findings were that Asian REITs became more inter-dependent during the 2009 Global Financial Crisis (GFC), but this has gradually declined since then. Moreover, developed Asian markets’ (Japan and Singapore) returns influence returns in emerging Asian markets, and both Singapore and Hong Kong are the sources of volatility spillovers to the rest of the Asian markets (Japan, Malaysia, Taiwan, Thailand, and South Korea). Using the asymmetric BEKK (Baba–Engle–Kraft–Kroner) GARCH model [19], Hoesli and Reka [31] find that the REIT markets of the United States, United Kingdom, and Australia influenced the volatility of global REIT markets between 1990 and 2010 (the global REIT index is taken from the EPRA/NAREIT database). Following the same methodology, Begiazi et al. [36] also provides evidence of the relatively strong integration of United States and Asian-Pacific REIT markets. Their results show bi-directional volatility linkages between the Americas and Asia Pacific as well as Europe and Asia Pacific over the period between 2006 and 2013. In the case of mean returns, the authors find that shocks in Asia-Pacific region affect Europe. They found no cross market effects between the United States and Europe in the case of both returns and volatility co-movements.

Other emerging papers adopt the Diebold and Yılmaz [20,21,27] framework such as Liow and Newell [22]. They calculate a volatility spillover index [20] generated from an asymmetric BEKK-GARCH model to examine the volatility interdependence between China, Hong Kong, and Taiwan with the United States under both crisis and non-crisis periods. (The full sample period is January 1995–December 2009, and the crisis episodes are the Asian Financial Crisis and the Global Financial Crisis.) In this case, the volatility spillover index aggregates the spillover effects of each of the four countries measured by the forecast error variance component for each country coming from shocks to another country in the system. They find that volatility interconnectedness was at its highest during the 2009 Global Financial Crisis (GFC) and that the United States alone was the source of almost all the volatility shocks transmitted during this period, as would be expected. Hong Kong had the second highest spillover effects given its relatively developed REIT market. (The return spillovers are captured by the coefficients of the BEKK-GARCH model and the results show evidence of bi-directional return spillover effects between the US and Hong Kong. The US spillover effects to China are smaller.) To the best of our knowledge, Liow and Newell [22] were the first to use the volatility spillover index [20] methodology to capture the transmission of volatility shocks across global REITs.

Recently, Liow and Huang [23] also followed this framework and adopted the extended Diebold and Yılmaz [21,27] spillover index to estimate the total, directional, net, and net-pairwise volatility connectedness in ten global REIT markets between 2004 and 2017. The connectedness indices are computed from a TGARCH Glosten et al. [37] specification of the conditional covariance matrix. Like Pham [14], Liow and Newell [22], and others, their evidence shows that the volatility connectedness from the United States to the rest of REIT markets in the sample was at its highest during the 2009 GFC; however, during the 2010 European debt crisis, European REIT markets were dominant transmitters of volatility shocks to the US. Over the full sample period, The United States is still the largest transmitter of volatility shocks to the rest of the global REIT markets followed by France. In summary, this paper adopts the Diebold and Yılmaz [21,27] volatility connectedness approach, and our paper differs from the above-mentioned paper because our dynamic volatility connectedness estimates are derived from a generalized impulse response function of a TVP–VAR model of Antonakakis et al. [28]. This approach overcomes the arbitrary selection of a rolling window size and the associated loss of observations, unlike in the case of Liow and Huang [23]. Moreover, we also estimate the return co-movement in REIT markets, which are dynamic in contrast to fixed parameter estimates provided in many studies in the literature.

## 3. Methodology

A widely used approach to trace and evaluate spillovers in a predetermined network is the connectedness approach proposed by Diebold and Yılmaz [20,21,27]. In the seminal papers, the dynamics are estimated via a rolling-window VAR approach, which faces some drawbacks such as (i) outliers sensitivity, (ii) arbitrarily chosen rolling-window sizes, (iii) loss of observations, and (iv) the inability to analyze low-frequency datasets. Employing a TVP-VAR based connectedness framework—which is used in this study—overcomes those shortcomings, as is intensively discussed in Antonakakis et al. [28]. In particular, we estimate the following TVP-VAR(1) model as suggested by the Bayesian information criterion (BIC), which can be outlined as follows,

(1) $$\begin{array}{ccc} z_{t}= & B_{t}z_{t-1}+u_{t}\; & u_{t}∼N\left(0,S_{t}\right) \end{array}$$

(2) $$\begin{array}{ccc} vec(B_{t})= & vec\left(B_{t-1}\right)+v_{t}\; & v_{t}∼N\left(0,R_{t}\right) \end{array}$$

where $z_{t}$, $z_{t-1}$ and $u_{t}$ are k×1 dimensional vector, and $B_{t}$ and $S_{t}$ are k×k dimensional matrices. $vec(B_{t})$ and $v_{t}$ are $k^{2}\times 1$ dimensional vectors, whereas $R_{t}$ is a $k^{2}\times k^{2}$ dimensional matrix.

In a further step, we are calculating the *H*-step-ahead (scaled) generalized forecast error variance decomposition (GFEVD) introduced by Koop et al. [25] and Pesaran and Shin [26]. Notably, the GFEVD is completely invariant of the variable ordering opposed to the orthorgonalized forecast error variance decomposition (see [20]). (We want to stress that even though we are talking about the spillovers of shocks, we are well aware that this interpretation differs from the macroeconomic literature; however, with this interpretation we are just following the interpretations Diebold and Yılmaz [20,21,27] to be in line with the connectedness literature.) We have decided to apply the GFEVD approach, as—to the best of our knowledge—no economic theory is developed that determines the structure of sectoral shocks. Hence, choosing an arbitrary error structure will lead to unreasonable results, and thus a GFEVD framework should be preferred [38]. Since this concept requires transforming the TVP–VAR into a TVP–VMA model, we make use of the Wold representation theorem: $z_{t}=\sum_{i=1}^{p}B_{it}z_{t-i}+u_{t}=\sum_{j=0}^{\infty }A_{jt}u_{t-j}$.

The (scaled) GFEVD (${\tilde{\phi }}_{ij,t}^{g}\left(H\right)$) normalizes the (unscaled) GFEVD ($\phi_{ij,t}^{g}\left(H\right)$) so that each row sums up to unity. ${\tilde{\phi }}_{ij,t}^{g}\left(H\right)$ represents the influence variable *j* has on variable *i* in terms of its forecast error variance share which is defined as the *pairwise directional connectedness from j to i*. This indicator is computed by

$$\begin{array}{c} \phi_{ij,t}^{g}\left(H\right)=\frac{S_{ii,t}^{-1}\sum_{t=1}^{H-1}(\iota_{i}^{\prime }A_{t}S_{t}\iota_{j}{)}^{2}}{\sum_{j=1}^{k}\sum_{t=1}^{H-1}(\iota_{i}A_{t}S_{t}A_{t}^{\prime }\iota_{i})}\;\;{\tilde{\phi }}_{ij,t}^{g}\left(H\right)=\frac{\phi_{ij,t}^{g}\left(H\right)}{\sum_{j=1}^{k}\phi_{ij,t}^{g}\left(H\right)} \end{array}$$

with $\sum_{j=1}^{k}{\tilde{\phi }}_{ij,t}^{g}\left(H\right)=1$, $\sum_{i,j=1}^{k}{\tilde{\phi }}_{ij,t}^{g}\left(H\right)=k$, and $\iota_{j}$ corresponds to a selection vector with unity on the *j*th position and zero otherwise.

Based upon the GFEVD, Diebold and Yılmaz [21,27] derived their connectedness measures. As mentioned previously, ${\tilde{\phi }}_{ij,t}^{g}\left(H\right)$ illustrates the impact a shock in variable *j* has on variable *i*. First, we present the *total directional connectedness to others*, which is the aggregated impact a shock in variable *j* has on all other variables, while the *total directional connectedness from others* illustrates the aggregated influence all other variables have on variable *j*. Those measures can mathematically be formulated as follows,

(3) $$\begin{array}{cc} TO_{jt}= & \sum_{i=1,i\neq j}^{k}{\tilde{\phi }}_{ij,t}^{g}\left(H\right) \end{array}$$

(4) $$\begin{array}{cc} FROM_{jt}= & \sum_{i=1,i\neq j}^{k}{\tilde{\phi }}_{ji,t}^{g}\left(H\right) \end{array}$$

Subtracting the impact that variable *j* has on others by the influence that others have on variable *j* results in the *net total directional connectedness*, which provides information about whether a variable is a net transmitter or a net receiver of shocks. Variable *j* is a net transmitter (receiver) of shocks— hence driving (driven by) the network—if the impact variable *j* has on others is larger (smaller) than the influence all others have on variable *j*, $NET_{jt}>0$ ($NET_{jt}<0$):

(5) $$\begin{array}{cc} NET_{jt}= & TO_{jt}-FROM_{jt} \end{array}$$

Another essential connectedness measure represents the corrected *total connectedness index ($TCI_{t}$)* [39,40], which can be described as the average impact one variable has on all others. If this measure is relatively high, it implies that the interconnectedness of the network and hence the market risk is high as well and vice versa. The corrected TCI can be computed by,

(6) $$\begin{array}{cc} TCI_{t}= & {\left(k-1\right)}^{-1}\sum_{j=1}^{k}TO_{jt}\equiv k^{-1}\sum_{j=1}^{k}FROM_{jt}. \end{array}$$

Since all aforementioned measures offer information on an aggregated basis, the *net pairwise directional connectedness ($NPDC_{ji,t}$)* tells us more about the bilateral relationship between variable *j* and *i*. This measure exhibits whether variable *i* is driving or driven by variable *j*. Therefore, we subtract the impact variable *i* has on variable *j* from the influence variable *j* has on variable *i*. If $NPDC_{ji,t}>0$ ($NPDC_{ji,t}<0$), it means that variable *j* is dominating (dominated by) variable *i*:

(7) $$\begin{array}{cc} NPDC_{ji,t}= & {\tilde{\phi }}_{ij,t}\left(H\right)-{\tilde{\phi }}_{ji,t}\left(H\right) \end{array}$$

## 4. Data

The international REIT return and volatility spillovers are examined on twelve developed REIT markets with the data sourced from the S&P Global REIT series in the Bloomberg database. The indices are daily closing prices from 1 October 2007 to 25 May 2021 and are all measured in US dollars. The countries are the United States, Canada, United Kingdom, France, Germany, Belgium, Netherlands, Japan, Hong Kong, Singapore, Australia, and New Zealand, spanning four continents or regions.

**Figure 1:**
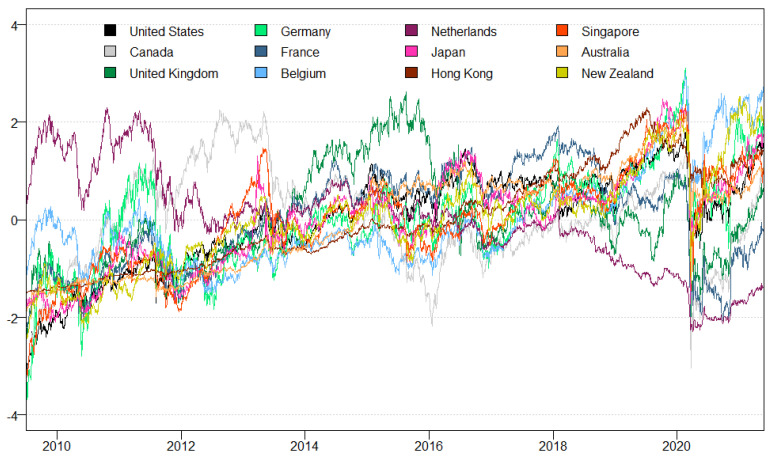
Real Estate Investment Trust Series.

Table 1 presents the descriptive statistics of the dataset. The Netherlands on average has the largest returns, calculated in log differences, while the rest of the markets or countries have negative returns. France, Germany, the UK, and the Netherlands have relatively larger deviations from their respective means when looking at variances, while the rest of the countries do not exhibit large variability. The skewness coefficients show that most of series are positively skewed, and in particular, Canada shows rather large deviations from a normal distribution. The coefficients of the Jarque-Bera test indicate that all the time-series are not normally distributed. Unit root tests are estimated based on the Stock et al. [41] test, and the test statistics indicate that the null hypothesis of non-stationarity can be rejected for all countries except the US. The sample of countries represents all *established* and *mature* REIT markets. Figure 1 plots a time series of REIT returns in log differences. The returns are broadly within a given range and show no outliers. REIT returns in Belgium, New Zealand and Germany showed a stronger recovery after the initial Covid-19 shock in 2020.

## 5. Empirical Results

Table 2 and Table 3 shows the “input–ouput” decomposition of the average total return and volatility connectedness or spillover index for the full sample. In Table 3, the absolute returns are used as a proxy for volatility connectedness. The *ij*-th value in the table represents the estimated contribution to the forecast error variance of country *i* coming from innovations or shocks to country *j*. The off diagonal column sums labelled as (Contributions to others) and the row sums labelled as (Contribution from the others) measure the *to* and *from* directional spillovers. Directional spillovers decompose the total spillover index into spillovers coming *from* and *to* a particular source. The net spillover is a simple difference between the *to* and *from* directional spillovers and summarises, in net terms, how much each country contributes to spillovers in other countries. Furthermore, the net pairwise spillovers is the difference between spillovers transmitted between a pair of individual countries *i* and *j* and vice versa. The total spillover index measures the contribution of spillover shocks across all countries to the total forecast error variance and is shown in the bottom far right of the table as “TCI”. It is calculated as the off-diagonal column sum or row sum totaled across all the countries over the column sum or row sum, including the diagonals totaled across all countries, and is expressed as a percentage.

In Table 2, the total connectedness index (TCI) for returns is 64.8% larger than the volatility TCI of 58.9% in Table 3, but shows a high degree of global REIT market interdependence. The return TCI is lower than the 71.1% total spillover index from Liow and Huang [23] and much higher than the 23.9% estimate from Liow and Newell [22]. The US (23.8%) is the dominant net transmitter of return spillovers followed by the European countries, France (18.9%), the Netherlands (15.8%), and to a lesser extent Belgium (8.55%). Asia-Pacific countries are generally net receivers of return spillovers (Japan, Hong Kong, Singapore, and New Zealand), with Hong Kong being the most sensitive to return spillovers from others with an estimate of −21.9%. This is consistent with evidence from Zhou [33] and Pham [14], which showed that return and volatility shocks in larger and more developed REIT markets spillover to less developed REIT markets. In Asia-Pacific, however, Australia dominates other REIT markets in the region as it is the only net transmitter of both volatility and return shocks. Hoesli and Reka [31] also found Australia’s REITs to be globally influential in the international REIT market. In contrast to its European peers, Germany is a net receiver of return spillovers but only to a smaller extent relative to Asian-Pacific countries.

Table 3 reports the average spillover table for volatility proxied by absolute returns. The results are broadly similar to the return connectedness results. The US contributes in net terms the most volatility to others (19.4%), and the magnitude is notably larger than that of France (16.0%) and the Netherlands (9.4%). Canada, the UK, and Germany are also net transmitters of volatility shocks; however, their magnitudes are very small. The results in both Table 2 and Table 3 highlight the influential role of US REITs and to a lesser degree European REIT markets in global REIT markets. The US as a source of systemic risk in international REITs has also been documented in other papers; see Liow and Jeongseop [45]. The relatively influential role of European REITs in our sample could possibly also reflect the impact of the European Sovereign Debt Crisis on financial markets, including REITs, as the crisis intensified from 2010 onwards.

Table 4 and Table 5 show the static return and absolute return connectedness results. Evidence from tables show that US and European REIT are broadly net transmitters of shocks while Asian-Pacific REITs are net recievers. THis is both returns and absloute returns cases and affirms evidence of the dynamic results in Table 2 and Table 3.

**Figure 2:**
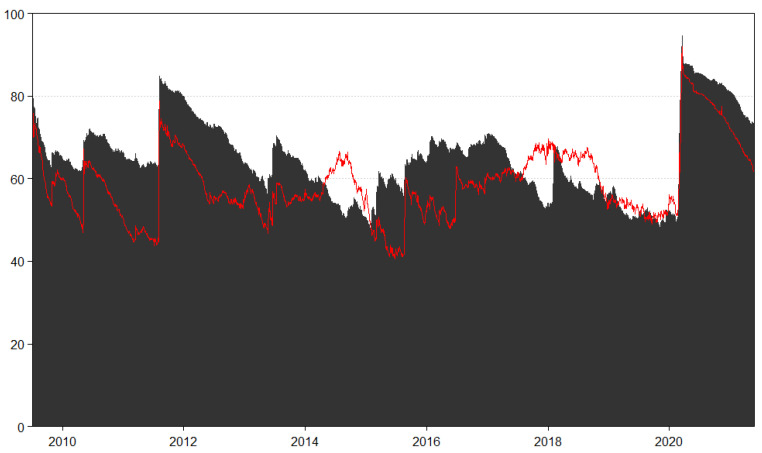
Dynamic Total Connectedness. Notes: Results are based on a TVP-VAR model with lag length of order one (BIC) and a 20-step-ahead generalized forecast error variance decomposition. Black area represents REIT return connectedness measures, while red line illustrates REIT volatility connectedness measures.

The spillover estimates in Table 2 and Table 3, while providing useful information, are static over the sample time horizon. We therefore estimate time-varying TCI for both returns and volatility in Figure 2 to capture how the transmission of shocks across REIT markets has evolved over time. In Figure 2, the black area shows the returns TCI and the volatility TCI is represented by the red line. Evidently, REIT market connectedness is a crisis sensitive in both return and volatility cases. In Figure 2, both return and volatility connectedness across REIT markets reached over 80% during crisis episodes. This is notable during the 2012 European Sovereign Debt (ESD) Crisis and more recently the COVID-19 Pandemic in 2020. Return and volatility connectedness reached closer to 100% at the peak of pandemic in March 2020 and were higher than the levels seen during the 2008 Global Financial Crisis, when connectedness rose to just above 90% based on evidence from Liow and Huang [23]. Although connectedness declined, it remained generally elevated into 2021 due to the prolonged uncertainty over the possibility of multiple wave of COVID-19 infections. Recent evidence from Periola-Fatunsin et al. [46], which focused on Asian REITs, also found high connectedness in both return and volatility as a result of the COVID-19 pandemic induced uncertainty shock. The impact of the COVID-19 pandemic on REITs was large with real estate sub-sectors such as retail and hospitality affected the most due to changes in consumer demand and spending patterns (e.g., the shift towards online shopping and travel restrictions) as a result of social distancing measures. Moreover, the effects could become long-term or lead to paradigm shifts in the real estate sector if changes in consumer spending behavior as a result of the evolving pandemic is permanent. In both the return and volatility cases, spillovers also increase in 2016, although the magnitude is lower and likely reflects uncertainty related to the Brexit bote and the US presidential election race.

**Figure 3:**
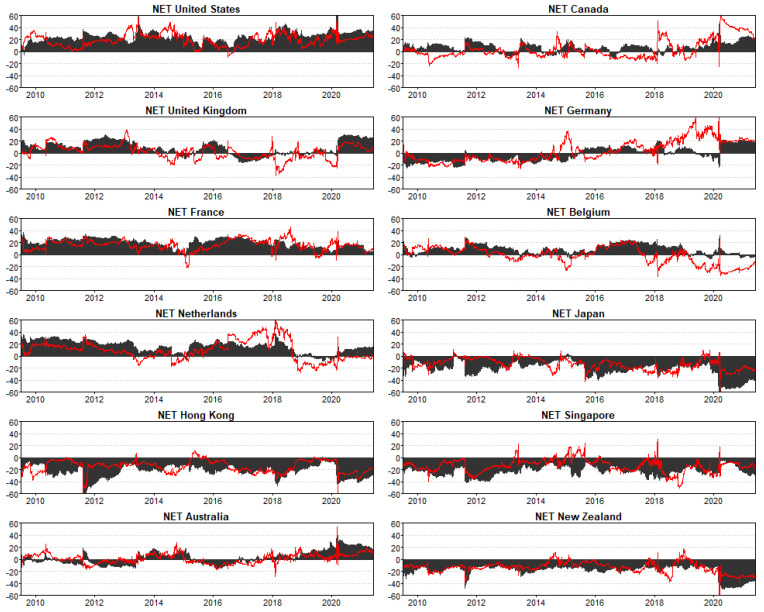
Net Total Directional Connectedness. Notes: Results are based on a TVP-VAR model with lag length of order one (BIC) and a 20-step-ahead generalized forecast error variance decomposition. Black area represents REIT return connectedness measures, and red line illustrates REIT volatility connectedness measures.

Figure 3 shows the dynamic net directional spillovers: the net transmitters are indicated by positive implied volatility estimates, and the negative estimates show that the REIT market or country is a receiver of volatility shocks. The black shaded area represents returns, and volatility is shown by the red line. In most cases, return and volatility connectedness move in tandem. Notably, Asian-Pacific countries of Japan, Hong Kong, Singapore, and New Zealand are permanent net receivers of return and volatility shocks, even during the COVID-19 pandemic, which originated in the Asia-Pacific region. Evidence in this paper contradicts findings by Periola-Fatunsin et al. [46], who found that Japan and Singapore were net transmitters of volatility spillovers during the COVID-19 outbreak. The US is a consistent net giver of return and volatility shocks, and the magnitude of spillovers are relatively large followed by France, Netherlands, and Belgium. The latter markets are relatively less risky from a portfolio diversification perspective given that their sources of cross-market risk are relatively few and or non-existent compared to countries that are mostly net receivers of volatility shocks. Figure 3 also shows that US REITs were mostly the source of shocks during the outbreak of the first wave of COVID-19 pandemic shown by a brief but sizable spike in spillovers relative to all other countries in the sample.

**Figure 4:**
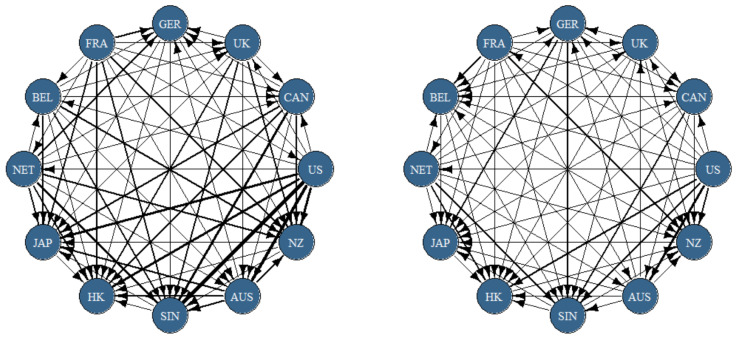
Net Pairwise Directional Connectedness. Notes: Results are based on a TVP-VAR model with lag length of order one (BIC) and a 20-step-ahead generalized forecast error variance decomposition. Black area represents REIT return connectedness measures, and red line illustrates REIT volatility connectedness measures.

Figure 4 presents the dynamic net pairwise spillovers. The black lines and arrows show the direction of the spillovers, and the bolder the line the stronger pairwise relationship. In Figure 4, the network plots show there are relatively strong return linkages between United States and Asian Pacific (Japan, Singapore, Hong Kong and Australia). The United States a a transmitter of return shocks to these countries. Canada also transmits return shocks to REIT markets in Singapore, Hong Kong, and Japan. These results highlight how REIT markets in North America and Asia Pacific are interconnected and that the relationship is mostly unidirectional with North America being a source of volatility shocks to Asia Pacific. Germany is also a net receiver of return shocks from others, particularly other European countries. One possible reason is the relatively small size of the German REIT market, and underlying this is possibly due to the exclusion residential property in German REITs by German REIT legislation, which has discouraged investors from choosing German REITs and therefore constrained the growth of the German REIT market (Newell and Marzuki [47]).

The right-hand network plot in Figure 4 shows the pairwise net directional volatility connectedness. The volatility connectedness in this case is not as strong as the case of returns, and this is similar to the results in Table 2 and Table 4, which show a larger return TCI value compared to the volatility TCI. European REITs are also net givers of both return and volatility to Asia-Pacific countries, in contrast to evidence by Periola-Fatunsin et al. [46], who found that Asia-Pacific shocks affected the European mean returns and that this relationship was unidirectional. In Europe, Belgium receives volatility shocks mostly from other European countries, while France is not affected by spillovers from others except the US in the case of volatility. The US also dominates Europe REITs in both the left- and right-hand network plots. Overall, the evidence shows a strong market integration between REIT markets of Europe and Asia-Pacific as well as North America and Asia-Pacific. REIT markets in the US, and to a lesser extent France, offer opportunities for diversification and the mitigation of cross-market risk for investors because they are generally not vulnerable to receive shocks from other REIT markets.

## 6. Concluding Remarks

Real estate investment trusts have grown substantially in terms of their global market capitalization and have become an important asset class in investment portfolios. This paper studies systemic risk and the transmission of shocks in international REIT markets because empirical evidence in this regard is scant. We adopted the TVP–VAR-based connectedness framework of Antonakakis et al. [28], which has several advantages over theDiebold and Yılmaz [21,27] framework, to construct time-varying total, net and pairwise return and volatility connectedness measures for a representative sample of international REIT markets spanning North America, Asia Pacific and Europe. Our findings show that both returns and volatility spillovers are dynamic and that connectedness increased sharply during times of market distress, indicating reduced diversification benefits during such events. We find that the total volatility and return connectedness averaged 58.9% and 64.8%, suggesting that global REIT interdependence is greater in the case of returns compared to volatility spillovers. The US REITs are the main source of volatility and return spillovers to others followed by European REITs. The dominant role of US REITs in part reflects its maturity and relative size in the international REIT market, making up 66.4% of the FTSE EPRA/NAREIT Global real estate index (EPRA (2020), Global REIT Survey, European Public Real Estate Association, Brussels). Asian-Pacific REITs are generally net receivers of shocks, with the exception of Australia, possibly reflecting the shared characteristics such as geographical proximity and relatively nascent development. However, this could change as markets continue to develop and evolve. These findings contribute to the emergent literature and improve our understanding of the dynamics of shocks in REIT markets and how these are transferred across other REIT markets. Finally, due to the continuously growing global financial market integration as well as cross-listing of REITs across regions, monitoring of these cross market linkages within the REIT asset class will become increasingly important for portfolio risk management purposes.

## Figures and Tables

**Table 1:** Summary Statistics.

	United States	Canada	United Kingdom	Germany	France	Belgium	Netherlands	Japan	Hong Kong	Singapore	Australia	New Zealand
Mean	−0.026	−0.015	−0.006	−0.011	−0.005	−0.012	0.035	−0.012	−0.055	−0.016	−0.024	−0.021
Variance	2.048	1.437	2.432	2.824	2.977	1.409	2.572	1.643	1.117	0.708	0.993	1.199
Skewness	1.871 ***	3.084 ***	2.245 ***	0.487 ***	0.591 ***	1.247 ***	0.889 ***	2.222 ***	0.238 ***	1.202 ***	2.139 ***	2.229 ***
	(0.000)	(0.000)	(0.000)	(0.000)	(0.000)	(0.000)	(0.000)	(0.000)	(0.000)	(0.000)	(0.000)	(0.000)
Excess	29.907 ***	51.308 ***	37.833 ***	11.544 ***	18.992 ***	16.315 ***	12.376 ***	55.006 ***	5.733 ***	25.105 ***	37.166 ***	39.160 ***
Kurtosis	(0.000)	(0.000)	(0.000)	(0.000)	(0.000)	(0.000)	(0.000)	(0.000)	(0.000)	(0.000)	(0.000)	(0.000)
JB	117566 ***	345617 ***	187845 ***	17370 ***	46863 ***	35253 ***	20232 ***	394131 ***	4282 ***	82316 ***	181134 ***	201028 ***
	(0.000)	(0.000)	(0.000)	(0.000)	(0.000)	(0.000)	(0.000)	(0.000)	(0.000)	(0.000)	(0.000)	(0.000)
ERS	−1.131	−4.372 ***	−2.900 ***	−2.941 ***	−3.375 ***	−4.565 ***	−9.249 ***	−4.113 ***	−2.456 **	−7.732 ***	−2.885 ***	−2.353 **
	(0.258)	(0.000)	(0.004)	(0.003)	(0.001)	(0.000)	(0.000)	(0.000)	(0.014)	(0.000)	(0.004)	(0.019)
Q(10)	81.136 ***	52.966 ***	43.957 ***	21.450 ***	41.177 ***	30.530 ***	85.457 ***	90.253 ***	12.751 **	71.646 ***	83.743 ***	32.198 ***
	(0.000)	(0.000)	(0.000)	(0.000)	(0.000)	(0.000)	(0.000)	(0.000)	(0.018)	(0.000)	(0.000)	(0.000)
$Q^{2}\left(10\right)$	931.729 ***	1492.857 ***	266.989 ***	773.091 ***	883.458 ***	914.616 ***	799.925 ***	877.982 ***	242.531 ***	2816.581 ***	797.843 ***	423.605 ***
	(0.000)	(0.000)	(0.000)	(0.000)	(0.000)	(0.000)	(0.000)	(0.000)	(0.000)	(0.000)	(0.000)	(0.000)
	United States	Canada	United Kingdom	Germany	France	Belgium	Netherlands	Japan	Hong Kong	Singapore	Australia	New Zealand
United States	1.000	0.365	0.244	0.198	0.265	0.205	0.244	0.022	0.059	0.090	0.580	0.115
Canada	0.365	1.000	0.297	0.240	0.297	0.262	0.284	0.071	0.108	0.157	0.203	0.225
United Kingdom	0.244	0.297	1.000	0.380	0.536	0.455	0.511	0.072	0.136	0.182	0.171	0.250
Germany	0.198	0.240	0.380	1.000	0.426	0.437	0.425	0.083	0.099	0.150	0.115	0.202
France	0.265	0.297	0.536	0.426	1.000	0.538	0.644	0.068	0.111	0.164	0.185	0.229
Belgium	0.205	0.262	0.455	0.437	0.538	1.000	0.546	0.102	0.108	0.168	0.117	0.244
Netherlands	0.244	0.284	0.511	0.425	0.644	0.546	1.000	0.084	0.120	0.189	0.153	0.235
Japan	0.022	0.071	0.072	0.083	0.068	0.102	0.084	1.000	0.068	0.103	0.036	0.133
Hong Kong	0.059	0.108	0.136	0.099	0.111	0.108	0.120	0.068	1.000	0.242	0.055	0.109
Singapore	0.090	0.157	0.182	0.150	0.164	0.168	0.189	0.103	0.242	1.000	0.078	0.156
Australia	0.580	0.203	0.171	0.115	0.185	0.117	0.153	0.036	0.055	0.078	1.000	−0.073
New Zealand	0.115	0.225	0.250	0.202	0.229	0.244	0.235	0.133	0.109	0.156	−0.073	1

Notes: ***, **, * denote significance at 1%, 5% and 10% significance level; Skewness: D’Agostino [42] test; Kurtosis: Anscombe and Glynn [43] test; JB: Jarque and Bera [44] normality test; and ERS: Stock et al. [41] unit-root test.

**Table 2:** Averaged Return Connectedness Table.

	United States	Canada	United Kingdom	Germany	France	Belgium	Netherlands	Japan	Hong Kong	Singapore	Australia	New Zealand	FROM Others
United States	35.53	11.44	5.47	3.92	6.37	4.52	5.38	0.52	0.75	1.80	22.29	2.01	64.47
Canada	13.28	39.87	6.85	5.28	6.95	5.80	6.41	1.05	1.20	2.79	5.75	4.78	60.13
United Kingdom	5.97	6.00	29.22	8.42	14.63	11.30	13.27	0.71	1.13	2.19	3.82	3.33	70.78
Germany	4.93	5.43	9.47	33.55	11.90	12.79	12.01	0.85	0.83	2.15	2.88	3.22	66.45
France	5.89	5.64	13.02	9.41	25.66	13.59	16.75	0.72	0.78	1.84	3.55	3.14	74.34
Belgium	4.98	5.24	11.03	11.04	14.83	28.21	14.57	0.86	0.73	2.14	2.75	3.61	71.79
Netherlands	5.46	5.31	12.15	9.66	17.24	13.55	26.65	0.75	0.82	2.10	3.07	3.23	73.35
Japan	3.87	3.59	2.54	2.66	2.72	2.88	3.17	68.17	1.37	2.68	3.25	3.10	31.83
Hong Kong	4.48	4.15	3.44	2.17	3.32	2.37	2.95	1.46	61.55	8.41	3.14	2.58	38.45
Singapore	6.81	6.73	5.14	3.82	4.72	4.31	5.20	1.36	6.57	47.45	4.56	3.34	52.55
Australia	27.27	5.90	4.20	2.50	4.59	2.77	3.46	0.83	0.90	2.09	43.64	1.85	56.36
New Zealand	5.29	7.43	5.71	5.02	5.99	6.46	5.97	1.96	1.50	2.82	4.24	47.60	52.40
TO others	88.24	66.87	79.00	63.90	93.27	80.34	89.15	11.08	16.58	31.01	59.29	34.19	TCI
NET	23.76	6.74	8.22	−2.56	18.93	8.55	15.80	−20.76	−21.87	−21.54	2.93	−18.21	64.81

Notes: Results are based on a TVP–VAR model with lag length of order one (BIC) and a 20-step-ahead generalized forecast error variance decomposition.

**Table 3:** Averaged Absolute Return Connectedness Table.

	United States	Canada	United Kingdom	Germany	France	Belgium	Netherlands	Japan	Hong Kong	Singapore	Australia	New Zealand	FROM Others
United States	39.69	8.95	4.77	5.04	5.34	3.47	4.61	1.70	1.15	3.71	18.89	2.68	60.31
Canada	10.45	45.53	5.31	5.98	6.14	4.36	4.89	2.29	1.37	3.90	5.49	4.28	54.47
United Kingdom	5.84	5.09	37.05	7.01	13.02	8.95	11.17	1.79	1.16	2.60	3.41	2.91	62.95
Germany	6.30	5.77	7.29	40.61	9.80	9.33	8.63	1.80	0.93	2.62	3.57	3.35	59.39
France	5.46	5.09	11.23	8.31	31.06	11.55	16.24	1.52	1.10	2.60	3.16	2.68	68.94
Belgium	4.86	4.41	9.16	9.51	13.70	34.89	12.68	1.41	0.99	2.50	3.25	2.64	65.11
Netherlands	5.31	4.27	9.98	7.62	17.14	11.24	34.04	1.07	1.00	2.85	2.93	2.55	65.96
Japan	3.69	3.95	3.19	3.91	3.39	2.59	2.01	66.53	1.45	3.45	3.23	2.63	33.47
Hong Kong	3.65	3.10	2.35	2.05	3.04	2.09	3.06	1.88	69.16	4.74	2.73	2.16	30.84
Singapore	6.34	6.20	4.18	4.79	4.60	3.44	5.01	2.81	3.76	51.18	4.07	3.62	48.82
Australia	22.89	5.88	3.35	3.51	3.58	2.63	3.10	1.83	0.96	2.91	47.31	2.06	52.69
New Zealand	4.88	5.81	4.46	5.25	5.19	3.98	3.94	2.21	2.21	3.72	3.44	54.92	45.08
TO others	79.66	58.50	65.27	62.97	84.93	63.62	75.35	20.31	16.08	35.59	54.17	31.58	TCI
NET	19.35	4.03	2.32	3.59	15.99	−1.49	9.39	−13.17	−14.76	−13.23	1.48	−13.50	58.91

Notes: Results are based on a TVP–VAR model with lag length of order one (BIC) and a 20-step-ahead generalized forecast error variance decomposition.

**Table 4:** Static Return Connectedness Table.

	United States	Canada	United Kingdom	Germany	France	Belgium	Netherlands	Japan	Hong Kong	Singapore	Australia	New Zealand	FROM Others
United States	28.63	12.85	6.28	4.88	7.74	5.54	7.07	0.57	0.63	2.94	20.78	2.09	71.37
Canada	13.95	30.09	7.23	6.30	8.19	6.37	7.26	0.94	1.25	4.89	8.38	5.15	69.91
United Kingdom	7.06	6.98	26.63	8.08	13.82	11.38	13.50	0.56	0.92	2.70	4.64	3.74	73.37
Germany	6.10	7.03	9.77	32.07	11.36	11.43	11.31	0.74	0.60	2.79	3.44	3.36	67.93
France	7.04	6.73	12.35	8.35	23.96	12.84	17.31	0.24	0.77	2.63	4.56	3.22	76.04
Belgium	6.19	6.00	11.41	9.42	14.33	26.52	14.77	0.66	0.55	2.93	3.60	3.62	73.48
Netherlands	6.97	6.20	12.14	8.37	17.55	13.25	24.06	0.53	0.74	2.76	4.26	3.17	75.94
Japan	4.69	5.28	2.84	3.79	3.80	3.11	3.95	60.73	0.91	4.73	4.40	1.77	39.27
Hong Kong	3.88	4.84	3.01	1.84	3.51	2.07	3.37	0.99	62.67	8.68	2.67	2.49	37.33
Singapore	8.21	8.85	5.01	4.44	5.85	5.34	6.43	2.68	5.19	37.70	6.43	3.87	62.30
Australia	25.88	9.65	4.97	3.42	6.17	3.75	5.00	1.01	0.73	3.59	35.74	0.11	64.26
New Zealand	7.19	9.58	6.35	5.08	6.40	6.51	6.57	1.78	1.44	4.23	3.71	41.17	58.83
TO others	97.16	83.97	81.34	63.97	98.74	81.58	96.55	10.69	13.72	42.85	66.87	32.58	TCI
NET	25.79	14.06	7.97	−3.96	22.70	8.11	20.61	−28.58	−23.61	−19.45	2.61	−26.25	64.17

Notes: Results are based on a VAR model with lag length of order one (BIC) and a 20-step-ahead generalized forecast error variance decomposition.

**Table 5:** Static Absolute Return Connectedness Table.

	United States	Canada	United Kingdom	Germany	France	Belgium	Netherlands	Japan	Hong Kong	Singapore	Australia	New Zealand	FROM Others
United States	31.87	10.92	4.26	4.52	6.33	4.68	5.74	1.71	0.62	5.78	19.96	3.59	68.13
Canada	12.01	34.47	4.53	5.08	7.08	4.59	5.05	2.94	1.00	7.97	9.13	6.16	65.53
United Kingdom	6.09	5.50	35.26	6.26	12.57	9.38	11.51	1.56	0.81	3.26	4.50	3.30	64.74
Germany	7.12	6.54	7.59	40.96	9.26	8.77	8.14	1.03	0.38	2.99	4.23	2.96	59.04
France	6.93	6.50	9.96	6.29	28.36	10.55	16.52	1.57	0.97	4.32	5.21	2.82	71.64
Belgium	6.80	5.20	9.18	6.84	12.67	32.43	12.44	1.41	0.70	3.98	5.44	2.92	67.57
Netherlands	6.88	4.86	9.97	5.83	17.88	11.19	30.50	0.82	0.90	4.11	4.81	2.25	69.50
Japan	5.37	10.91	2.85	3.38	3.88	2.31	2.02	52.49	0.92	6.15	4.66	5.06	47.51
Hong Kong	3.62	3.88	2.31	1.22	3.92	1.52	2.85	1.41	69.41	5.39	2.87	1.60	30.59
Singapore	9.19	11.13	3.76	3.80	6.17	4.08	5.08	4.68	2.47	36.09	7.80	5.77	63.91
Australia	22.81	9.82	3.48	3.24	5.04	3.83	4.47	2.03	0.62	5.65	36.00	3.02	64.00
New Zealand	6.13	9.87	4.53	4.29	6.30	4.47	3.43	3.58	0.92	6.75	4.74	44.97	55.03
TO others	92.95	85.13	62.41	50.77	91.09	65.37	77.26	22.74	10.30	56.36	73.36	39.44	TCI
NET	24.83	19.60	−2.33	−8.27	19.45	−2.20	7.77	−24.76	−20.29	−7.55	9.35	−15.59	60.60

Notes: Results are based on a VAR model with lag length of order one (BIC) and a 20-step-ahead generalized forecast error variance decomposition.
